# Machine learning for prediction of key haemodynamic parameters in pulmonary arterial hypertension

**DOI:** 10.1093/ehjdh/ztaf074

**Published:** 2026-06-18

**Authors:** Tilmann Kramer, Henning Weis, Mira Kramer, Stephan Baldus, Stephan Rosenkranz, Stefan Spinler

**Affiliations:** Department of Internal Medicine III, Heart Center at the University of Cologne, Kerpener Str. 62, D-50937 Cologne, Germany; Chair of Logistics Management (Kühne Foundation Endowed Chair), WHU—Otto Beisheim School of Management, D-56179 Vallendar, Germany; Department of Nuclear Medicine, University Hospital of Cologne, Cologne, Germany; Department of Anesthesiology and Intensive Care Medicine, Hospital Cologne-Merheim, Clinic for Anesthesiology and Operative Intensive Medicine, Cologne, North Rhine-Westphalia, Germany; Department of Internal Medicine III, Heart Center at the University of Cologne, Kerpener Str. 62, D-50937 Cologne, Germany; Cologne Cardiovascular Research Center (CCRC), Heart Center at the University of Cologne, Cologne, Germany; Department of Internal Medicine III, Heart Center at the University of Cologne, Kerpener Str. 62, D-50937 Cologne, Germany; Cologne Cardiovascular Research Center (CCRC), Heart Center at the University of Cologne, Cologne, Germany; Chair of Logistics Management (Kühne Foundation Endowed Chair), WHU—Otto Beisheim School of Management, D-56179 Vallendar, Germany

**Keywords:** Pulmonary arterial hypertension, Machine learning, Haemodynamic assessment, Non-invasive prediction, Right heart catheterization, Cardiopulmonary haemodynamics

## Abstract

**Aims:**

Machine learning (ML) is increasingly recognized for its ability to identify and structure variables for predictive tasks. Pulmonary arterial hypertension (PAH) is a progressive disease characterized by elevated mean pulmonary arterial pressure (mPAP) and pulmonary vascular resistance (PVR) with normal pulmonary arterial wedge pressure (PAWP), as assessed by right heart catheterization (RHC). Despite increased awareness, delays between onset of non-specific symptoms and diagnosis continue to hinder early initiation of targeted therapies, leading to poorer outcomes. To develop and evaluate ML models for predicting key haemodynamic parameters in PAH, based on routinely available non-invasive data collected within 8 weeks prior to RHC, as a proof of concept.

**Methods and results:**

We analysed data from 181 patients with invasively confirmed PAH, incorporating 56 variables, including demographics, echocardiography, blood gas analyses, 6-min walk distances, laboratory tests, and WHO functional class. An 80/20 train-test split and fivefold cross-validation were applied across multiple ML models, including least absolute shrinkage and selection operator (lasso) regression, ridge regression, k-nearest neighbours, decision trees, random forest, and gradient boosting machine. Lasso achieved best performance for predicting mPAP (*r* = 0.80, *R*² = 0.64, RMSE = 8.49). For PVR, ridge performed best (*r* = 0.71, *R*² = 0.51, RMSE = 3.60). Random forest and gradient boosting machines achieved modest but consistent performance for cardiac index (*r* = 0.38 and 0.37), while PAWP prediction remained limited across all models.

**Conclusion:**

Machine learning models can estimate mPAP and PVR from routine clinical data obtained prior to RHC in patients with confirmed PAH. External validation is required to confirm generalizability and clinical applicability.

## Graphical Abstract

**Graphical Abstract:**
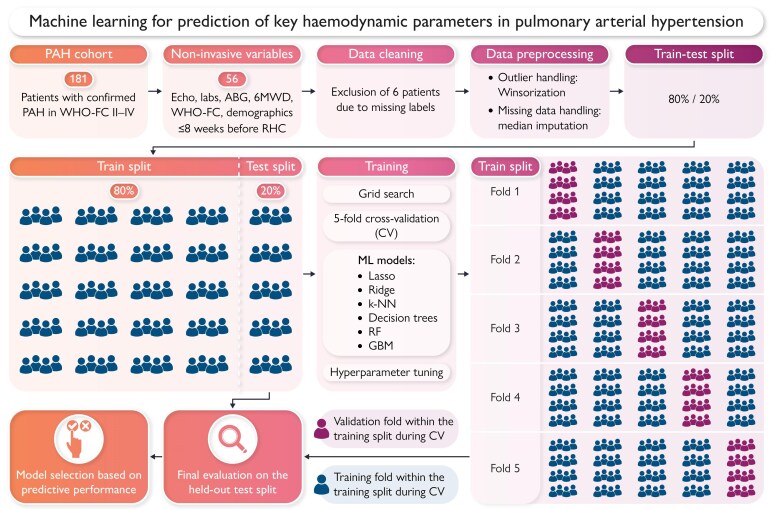

## Introduction

Pulmonary arterial hypertension (PAH) is a chronic, life-threatening disease defined by the 2022 ESC/ERS guidelines as mean pulmonary arterial pressure (mPAP) > 20 mmHg and pulmonary vascular resistance (PVR) > 2 Wood units (WU), with pulmonary arterial wedge pressure (PAWP) ≤ 15 mmHg, potentially leading to right heart failure and increased mortality.^1^ Diagnosis is confirmed by right heart catheterization (RHC), which remains the gold standard but is invasive, resource-intensive, and carries procedural risks, particularly outside experienced, high-volume centres.^2^ Over the past two decades, novel PAH therapies have been approved,^1^ significantly improving prognosis, increasing 1-year survival rates from 65% in the 1980s to around 90% and extending median survival from 2.8 to 6 years.^3,4^ Early PAH diagnosis remains challenging due to nonspecific symptoms like dyspnoea and fatigue.^1,5^ Diagnostic delays are linked to higher mortality rates and increased economic burden.^6,7^ Despite advancements in targeted therapies, the interval from symptom onset to diagnosis has not significantly shortened, depriving patients of essential therapies and leading to more advanced disease at diagnosis.^5,8^ Median diagnostic delay remains similar to the 1.27 years reported in the 1980s,^8^ with a mean delay of 2.5 years and approximately one-third of patients waiting over 2 years.^8^ Delays exceeding 2 years are associated with an 11% increase in mortality risk, even after adjusting for age, sex, and PAH subtype.^8^ Contributing factors include limited awareness among patients and physicians, highlighting the need for new screening strategies to achieve earlier diagnosis.^4,5^ Machine learning (ML) has shown potential for predicting pulmonary hypertension (PH) and distinguishing between PH due to left heart disease and PAH.^9–12^ However, no ML algorithm currently predicts haemodynamic severity using multiple non-invasive data sources prior to RHC.

## Methods

### Patients

We conducted a retrospective analysis of 181 patients with invasively confirmed PAH (Nice classification group 1; mPAP ≥25 mmHg, PVR >3 WU, PAWP ≤15 mmHg) according to the 2015 ESC/ERS guidelines, all presenting in WHO functional class (WHO-FC) II-IV between October 2010 and May 2022. Patients with mPAP 21–24 mmHg and PVR 2.1–3.0 WU were not considered, as these thresholds were introduced only in the 2022 guideline definition.^1^ Six patients with missing values in dependent variables were excluded, leaving 175 patients (114 female, 61 male) for final analysis. Data from 56 non-invasive clinical variables, including demographics, echocardiographic parameters, WHO-FC, 6-min walk distance (6MWD), and laboratory values, were collected within 8 weeks prior to RHC.

### Machine learning analysis

Data preprocessing and ML analysis were performed using R (v4.3.3, 2024, R Core Team) and RStudio (v2023.12, RStudio Team), applying multiple supervised models such as least absolute shrinkage and selection operator (lasso) regression, ridge regression, k-nearest neighbours (k-NN), decision trees, random forest (RF), and gradient boosting machine to predict key haemodynamic parameters.

Lasso was used for feature selection, applying L1 regularization to shrink less relevant coefficients to zero, thus reducing overfitting and improving model interpretability and efficiency by identifying only the variables with the highest predictive power. Ridge addressed multicollinearity by penalizing large coefficients without excluding them entirely, applying L2 regularization, which distributes the penalty across all coefficients. k-NN, a non-parametric algorithm, was used to evaluate proximity-based classification, capturing local data relationships for haemodynamic parameter prediction. Decision trees provided interpretable branching structures; however, to mitigate overfitting, RF, an ensemble method combining multiple decision trees, was used to enhance predictive accuracy and generalization. Gradient boosting machine builds models sequentially, with each iteration minimizing the residual error of the previous model and capturing complex data patterns.

### Data preprocessing

Winsorization at the 0.05 level replaced values below the 5th and above the 95th percentile with the respective percentile values. Median imputation addressed missingness in all non-dependent variables and was applied consistently across the dataset to align with the ML preprocessing pipeline. An 80/20 train-test split allocated 80% of the data for training and 20% for testing. Fivefold cross-validation was used to ensure robustness and prevent overfitting by dividing the training data into five subsets, iteratively validating on one while training on the remaining four. The average performance across all folds was used for model evaluation. Hyperparameter tuning optimized model outcomes.

### Model evaluation

Model performance was assessed using correlation coefficients (*r*) and coefficients of determination (*R*²), indicating the strength of association between predicted and actual values and the variance explained. Additionally, root mean squared error (RMSE), mean absolute error (MAE), and median absolute error were calculated to evaluate model accuracy. Models were primarily evaluated based on their ability to predict key haemodynamic indices, including mPAP, PVR, PAWP, and cardiac index (CI).

### Ethical considerations

Patients provided written informed consent. The study was approved by the Ethics Committee of the University of Cologne (22–1318_2-retro) and conducted in accordance with the Declaration of Helsinki.

## Results

### Baseline patient characteristics and right heart catheterization assessment

Patients had a mean age of 62.3 ± 16.7 years, with a female predominance (65.1%). Most patients (67.4%) were diagnosed with idiopathic PAH. Median *n*-terminal prohormone of brain natriuretic peptide (NT-proBNP) serum level was elevated at 1349 [interquartile range (IQR) 535–2629] ng/L, and mean 6MWD was reduced to 341 ± 107 m. Baseline RHC showed 46.3 ± 14.0 mmHg for mPAP, 10.5 ± 3.9 mmHg for PAWP, 10.0 ± 6.9 WU for PVR, and 2.2 ± 0.5 L/min/m^2^ for CI. Full baseline characteristics are provided in *Table 1*.

**Table 1:** Baseline characteristics of patients with newly diagnosed pulmonary arterial hypertension (*n* = 175), including demographics, pulmonary arterial hypertension subtype, comorbidities, functional status, haemodynamic, laboratory, and echocardiographic parameters

Demographics	*n* = 175
Age (years)	62.3 ± 16.7
Gender [f/m (%)]	114 (65.1)/61 (34.9)
Weight (kg)	77.9 ± 18.9
Height (cm)	168.3 ± 8.8
BMI (kg/m²)	26.9 (22.9–30.9)
BSA (m²)	1.9 ± 0.2

PAH subtype	*n* (%)
Idiopathic	118 (67.4)
Heritable	8 (4.6)
Drug-induced	4 (2.3)
Connective tissue disease	39 (22.3)
Congenital heart disease	3 (1.7)
HIV	2 (1.1)
Other	1 (0.6)

Comorbidities and cardiovascular risk factors	*n* (%)
Hypertension	114 (65.1)
Diabetes	37 (21.1)
Coronary artery disease	49 (28.0)
BMI > 30 kg/m^2^	50 (28.6)
Dyslipidaemia	49 (28.0)
History of atrial fibrillation	40 (22.9)
Functional status	
WHO-FC I [*n* (%)]	0 (0)
WHO-FC II [*n* (%)]	22 (12.6)
WHO-FC III [*n* (%)]	137 (78.3)
WHO-FC IV [*n* (%)]	16 (9.1)
6MWD (m)	341.0 ± 107.2
Haemodynamic parameters	
Systolic PAP (mmHg)	74.0 (54.8–88.0)
Diastolic PAP (mmHg)	27.2 ± 10.4
Mean PAP (mmHg)	46.3 ± 14.0
PAWP (mmHg)	10.5 ± 3.9
RAP (mmHg)	7.9 ± 3.8
CI (L/min/m^2^)	2.1 (1.8–2.5)
TPG (mmHg)	34.0 (23.0–46.0)
DPG (mmHg)	17.2 ± 10.9
SVI (mL/m^2^/beat)	28.2 ± 8.0
PVR (WU)	8.7 (5.2–12.1)
PA_C_ (mL/mmHg)	1.2 ± 0.6
SvO_2_ (%)	62.1 ± 7.8
Laboratory parameters	
NT-proBNP (ng/L)	1349 (535–2629)
Creatinine (mg/dL)	1.0 (0.9–1.2)
Echocardiographic parameters	
RA area (cm^2^)	23.0 (19.1–28.2)
RVEDD (mm)	43.1 (39.0–47.0)
PASP (mmHg)	78.5 (59.8–92.0)
TAPSE (mm)	17.7 ± 4.5
TAPSE/PASP (mm/mmHg)	0.2 (0.2–0.3)
LVEDD (mm)	41.9 ± 6.3
LVEF (%)	65.0 (60.0–65.0)

Data are presented as mean ± standard deviation (SD), median with interquartile range (IQR), or *n* (%), as appropriate for each variable.

6MWD, 6-min walk distance; BMI, body mass index; BSA, body surface area; CI, cardiac index; DPG, diastolic pulmonary pressure gradient; HIV, human immunodeficiency virus; Lasso, least absolute shrinkage and selection operator; LVEDD, left ventricular end-diastolic diameter; LVEF, left ventricular ejection fraction; MAE, mean absolute error; ML, machine learning; mPAP, mean pulmonary arterial pressure; NT-proBNP, *n*-terminal prohormone of brain natriuretic peptide; PAC, pulmonary arterial compliance; PAH, pulmonary arterial hypertension; PAP, pulmonary arterial pressure; PAWP, pulmonary arterial wedge pressure; PASP, pulmonary artery systolic pressure; PVR, pulmonary vascular resistance; *R*², coefficient of determination; RA, right atrium; RAP, right atrial pressure; *r*, correlation coefficient; RMSE, root mean squared error; RVEDD, right ventricular end-diastolic diameter; SvO₂, mixed venous oxygen saturation; TAPSE, tricuspid annular plane systolic excursion; TPG, transpulmonary pressure gradient; WHO-FC, World Health Organization functional class; WU, Wood units.

### Correlation analysis

**Figure 1:**
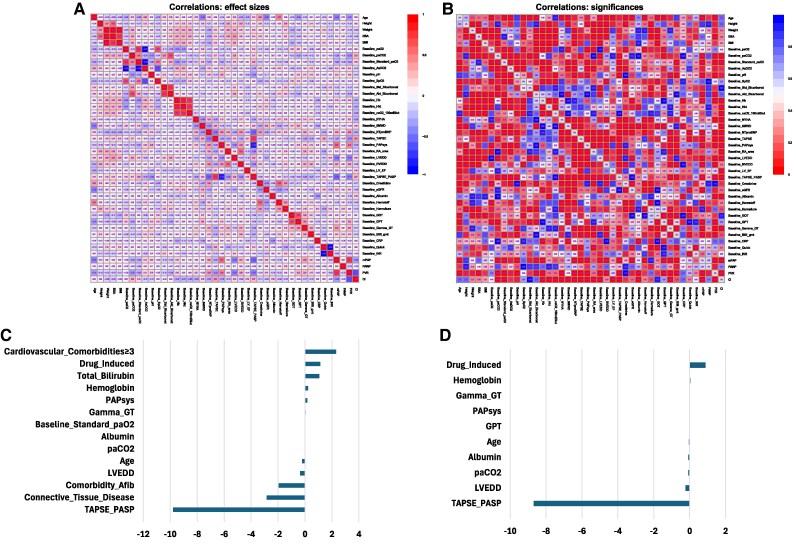
(*A*) Correlation heatmap illustrating the strength of association between all non-invasive baseline variables and the four key haemodynamic targets: mean pulmonary arterial pressure, pulmonary arterial wedge pressure, pulmonary vascular resistance, and cardiac index. (*B*) Corresponding heatmap of significance levels (*P*-values) for the same variable-target associations. (*C*) Effect sizes of variables selected by the lasso regression model for mean pulmonary arterial pressure prediction, based on validation using the independent test dataset. Variables not retained in the final model were shrunk to zero. (*D*) Effect sizes of variables selected for pulmonary vascular resistance prediction using the same lasso regression approach. Effect sizes represent the penalized regression coefficients of the final model, based on validation using the independent test dataset, and indicate the direction and relative strength of each variable’s contribution. AFib, atrial fibrillation; CI, cardiac index; Gamma-GT, gamma-glutamyltransferase; GPT, glutamate pyruvate transaminase; Lasso, least absolute shrinkage and selection operator; LVEDD, left ventricular end-diastolic diameter; mPAP, mean pulmonary arterial pressure; paCO₂, arterial partial pressure of carbon dioxide; paO₂, arterial partial pressure of oxygen; PAPsys, systolic pulmonary artery pressure; PAWP, pulmonary arterial wedge pressure; PVR, pulmonary vascular resistance; TAPSE/PASP, tricuspid annular plane systolic excursion to systolic pulmonary artery pressure ratio.

The correlation heatmap of effect sizes (*Figure 1A*) indicated multiple relationships among variables, suggesting multicollinearity. The heatmap of significance levels (*Figure 1B*) confirmed the statistical relevance of many associations. As ML algorithms can handle multicollinearity, no variables were aggregated or excluded.

### Model performance

Lasso performed best for predicting mPAP (*r* = 0.80; *R*²=0.64) and showed good results for PVR (*r* = 0.65; *R*²=0.42), though ridge slightly outperformed lasso for PVR (*r* = 0.71; *R*²=0.51). For CI, random forest and gradient boosting machine reached modest but consistent predictive performance (*r* = 0.38 and 0.37; *R*²=0.14 each). For PAWP, lasso performed best (*r* = −0.34; *R*²=0.12), though overall performance remained limited. Summary metrics (*r*, *R*², RMSE, MAE, and median absolute error) are provided in *Table 2* based on validation using the independent test dataset.

**Table 2:** Performance metrics for the prediction of mean pulmonary arterial pressure, pulmonary vascular resistance, pulmonary arterial wedge pressure, and cardiac index across different machine learning models

Model	Parameter	*r*	*R*²	RMSE	MAE	Median absolute error
Lasso regression						
	mPAP	0.80	0.64	8.49	6.88	6.74
	PVR	0.65	0.42	3.90	3.25	2.86
	PAWP	−0.34	0.12	3.15	2.65	2.39
	CI	0.30	0.09	0.49	0.44	0.45
Ridge regression						
	mPAP	0.74	0.55	9.20	7.92	8.46
	PVR	0.71	0.51	3.60	3.08	2.80
	PAWP	<0.01	<0.01	3.44	2.83	2.96
	CI	0.29	0.08	0.50	0.44	0.41
k-Nearest neighbours						
	mPAP	0.48	0.23	12.26	10.27	9.12
	PVR	0.46	0.21	4.48	3.50	2.72
	PAWP	−0.01	<0.01	3.19	2.62	2.72
	CI	0.27	0.07	0.50	0.42	0.45
Decision trees						
	mPAP	0.71	0.50	9.67	8.30	8.57
	PVR	0.35	0.12	4.78	4.11	3.41
	PAWP	NA	NA	3.12	2.64	2.44
	CI	NA	NA	0.52	0.45	0.52
Random forest						
	mPAP	0.76	0.57	9.15	7.44	7.00
	PVR	0.69	0.47	3.85	3.24	2.65
	PAWP	−0.28	0.08	3.37	2.82	2.68
	CI	0.38	0.14	0.49	0.43	0.47
Gradient boosting machine						
	mPAP	0.77	0.59	10.70	8.80	7.92
	PVR	0.68	0.46	4.26	3.60	3.41
	PAWP	−0.22	0.05	3.27	2.72	2.54
	CI	0.37	0.14	0.50	0.43	0.48

Reported metrics include *r*, *R*^²^, RMSE, MAE, and median absolute error.

6MWD, 6-min walk distance; BMI, body mass index; BSA, body surface area; CI, cardiac index; DPG, diastolic pulmonary pressure gradient; HIV, human immunodeficiency virus; Lasso, least absolute shrinkage and selection operator; LVEDD, left ventricular end-diastolic diameter; LVEF, left ventricular ejection fraction; MAE, mean absolute error; ML, machine learning; mPAP, mean pulmonary arterial pressure; NT-proBNP, *n*-terminal prohormone of brain natriuretic peptide; PAC, pulmonary arterial compliance; PAP, pulmonary arterial pressure; PAWP, pulmonary arterial wedge pressure; PASP, pulmonary artery systolic pressure; PVR, pulmonary vascular resistance; *R*², coefficient of determination; RA, right atrium; RAP, right atrial pressure; *r*, correlation coefficient; RMSE, root mean squared error; RVEDD, right ventricular end-diastolic diameter; SvO₂, mixed venous oxygen saturation; TAPSE, tricuspid annular plane systolic excursion; TPG, transpulmonary pressure gradient; WHO-FC, World Health Organization functional class; WU, Wood units.

### Key predictors and effect sizes

For mPAP, lasso identified a limited set of features with corresponding effect sizes, while all others were reduced to zero (*Figure 1C*). Among selected non-binary features, positive effect sizes included total bilirubin (1.06), haemoglobin (0.24), and gamma-glutamyl transferase (gamma-GT; 0.04); negative effect sizes for tricuspid annular plane systolic excursion to systolic pulmonary artery pressure ratio (TAPSE/PASP; −9.78) and left ventricular end-diastolic diameter (LVEDD; −0.36). For PVR, ridge performed best (*Table 2*) but retained more features due to L2 regularization. Given its comparable performance, lasso-based feature selection for PVR is shown in *Figure 1D*, illustrating the effect of L1 regularization and the resulting sparse, interpretable feature set. Positive effect sizes included haemoglobin (0.04), gamma-GT (0.013), systolic pulmonary artery pressure (PAPsys; 0.008), and glutamate pyruvate transaminase (GPT; 0.004); negative ones for TAPSE/PASP (−8.68) and LVEDD (−0.23).

## Discussion

Machine learning models, particularly lasso and ridge regression, predicted mPAP and PVR from non-invasive data prior to RHC with high accuracy. The study focused on patients with moderate to severe PAH, based on the previously applicable diagnostic thresholds, enabling robust pattern recognition. Lasso-based feature selection identified predictors of mPAP and PVR not typically emphasized in routine assessment, which may warrant further investigation. CI was predicted with modest but consistent performance across models, suggesting feasibility but highlighting the need for more data. In contrast, model performance for PAWP was limited, possibly due to PAWP’s susceptibility to measurement variability from volume shifts, intrathoracic pressures, and procedural factors. Identifying whether PAWP is > or ≤15 mmHg remains crucial for distinguishing pre- from post-capillary PH, with significant therapeutic implications in PAH.^1^ Previous ML studies using classification models based on non-invasive data reported an AUC of 0.98 for post-capillary PH prediction,^12^ and AUCs of 0.83 and 0.84 for PH and PAH, respectively .^9–11^ These approaches offer screening potential and support differentiation between pre- and post-capillary forms, potentially reducing unnecessary invasive and costly assessments .^9–12^

Diagnostic delays remain common in PAH, contributing to adverse outcomes and increased healthcare costs.^6–8^ Although our study does not directly address this issue, ML-based haemodynamic estimates could, after validation, help reduce time to diagnosis.

This proof-of-concept study highlights the potential of ML to support future workflows by identifying patients who may benefit from earlier RHC referral.

### Strengths and limitations

Strengths include precise haemodynamic diagnosis, comprehensive non-invasive data collection without clinical preselection, and a transparent preprocessing strategy. Machine learning model performance was evaluated on an independent test set, reducing overfitting risk. Missing data were handled by median imputation, preserving sample size and dataset consistency. Limitations include the retrospective, single-centre design, small sample size, lack of a control group, and a time interval of up to 8 weeks between data collection and RHC. Additionally, patients with mPAP 21–24 mmHg and PVR 2.1–3.0 WU, who would now meet the diagnostic criteria for PAH as defined by the 2022 ESC/ERS guidelines, were not considered, limiting generalizability to mild PAH. Still, the models demonstrated strong predictive performance within the defined cohort and data acquisition timeframe.

### Future directions

External validation in larger, more heterogeneous multicentre cohorts, including patients with mild PAH and other PH classes, are essential to confirm robustness. Shorter data collection intervals may further improve predictive precision. Integrating ML into clinical workflows requires Electronic health record (EHR)-compatible interfaces for real-time predictions, streamlined diagnostics, and timely referral and therapy initiation. Guidelines remain essential for diagnostic work-up.^1^ After further validation, ML could complement them by enabling individualized, data-driven predictions.

### Conclusions

This proof-of-concept study demonstrates that ML models can estimate haemodynamic severity in PAH using routine non-invasive data obtained prior to RHC. Validation in larger, more diverse cohorts is required to confirm generalizability and clinical utility.

## Data Availability

The data underlying this article will be shared on reasonable request to the corresponding author.
